# Reversible Immunosensor for the Continuous Monitoring of Cortisol in Blood Plasma Sampled with Microdialysis

**DOI:** 10.1021/acssensors.2c01358

**Published:** 2022-10-18

**Authors:** Laura van Smeden, Annet Saris, Khulan Sergelen, Arthur M. de Jong, Junhong Yan, Menno W. J. Prins

**Affiliations:** † Department of Biomedical Engineering, Eindhoven University of Technology, 5600 MBEindhoven, The Netherlands; ‡ Department of Applied Physics, Eindhoven University of Technology, 5600 MBEindhoven, The Netherlands; § Institute for Complex Molecular Systems (ICMS), Eindhoven University of Technology, 5600 MBEindhoven, The Netherlands; ∥ Helia Biomonitoring, De Lismortel 31, 5612 AREindhoven, The Netherlands

**Keywords:** continuous monitoring, single-molecule resolution, real time, microdialysis, tethered particle, affinity binder

## Abstract

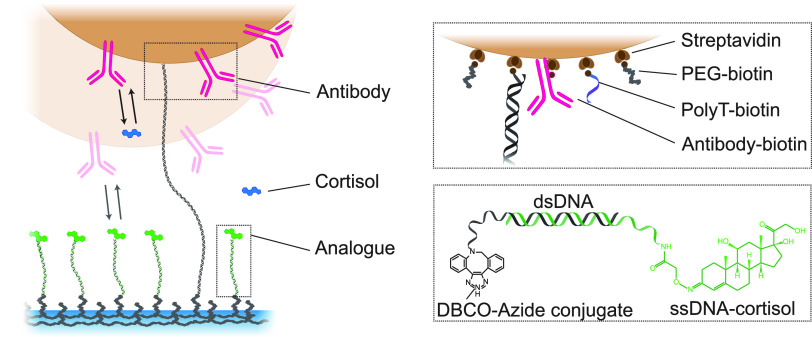

Cortisol is a steroid hormone involved in a wide range of medical conditions. The level of the hormone fluctuates over time, but with traditional laboratory-based assays, such dynamics cannot be monitored in real time. Here, a reversible cortisol sensor is reported that allows continuous monitoring of cortisol in blood plasma using sampling by microdialysis. The sensor is based on measuring single-molecule binding and unbinding events of tethered particles. The particles are functionalized with antibodies and the substrate with cortisol-analogues, causing binding and unbinding events to occur between particles and substrate. The frequency of binding events is reduced when cortisol is present in the solution as it blocks the binding sites of the antibodies. The sensor responds to cortisol in the high nanomolar to low micromolar range and can monitor cortisol concentrations over multiple hours. Results are shown for cortisol monitoring in filtered and in microdialysis-sampled human blood plasma.

Hormones are regulatory molecules that are transported through the body to control processes such as metabolism, growth and development, inflammation, emotions and mood, reproductive function, and sleep.^1−4^ Cortisol is a steroid stress hormone that fluctuates strongly and affects almost all tissues and organs in the body. Under healthy conditions, cortisol concentrations have a circadian profile^4−7^ with high concentrations in the morning (0.1–0.7 μM)^4−6^ and low concentrations during the night (0.1–0.4 μM).^4,6^ Elevated cortisol levels (above 0.7 μM) can result from chronic stress and relate to conditions such as heart disease, obesity, burnout, and Cushing’s syndrome.^1,8,9^ Monitoring cortisol–time profiles in individuals could aid in perioperative patient care and in the diagnosis and treatment of conditions with dysregulated or irregular cortisol levels such as Cushing’s syndrome.^1,5,10,11^

Sensors based on lateral-flow assays^12^ and electrochemical detection^13^ have been reported for the measurement of cortisol in biological fluids such as sweat, saliva, plasma, and serum.^7,12−19^ Cortisol–time profiles were recorded using separate sensors for every individual sample.^20^ In some studies, multiple samples were measured on a single sensor, as the cortisol concentration increased as a function of time.^21,22^ However, for measuring arbitrary cortisol–time profiles, a fully reversible sensor is needed that can record fluctuating cortisol concentrations with phases of increasing as well as decreasing cortisol concentration as a function of time.

A recently developed continuous biosensing technology called biosensing by particle mobility (BPM) is based on measuring reversible interactions between biofunctionalized particles and a biofunctionalized substrate.^23−29^ Previous BPM studies reported the monitoring of ssDNA, thrombin, and creatinine. Here, a BPM competition assay sensor is demonstrated for the monitoring of cortisol, using cortisol-analogues and anti-cortisol antibodies. We report measurements with multiple cycles of increasing and decreasing cortisol concentrations. Data are shown for cortisol in buffer, cortisol in filtered blood plasma, and cortisol sampled from blood plasma using a microdialysis catheter probe, as this represents an interfacing technology that is suitable for future patient monitoring.

## Results

### BPM Competition Immunoassay for Continuous Cortisol Monitoring

**Figure 1:**
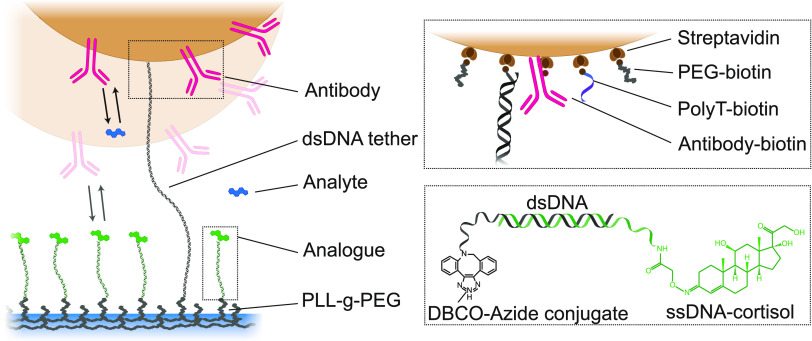
Design of a sensor for continuous cortisol monitoring based on biosensing by particle mobility, with a competition assay format. Streptavidin-coated particles (Dynabeads MyOne, ⌀1 μm) are functionalized with biotinylated anti-cortisol antibodies and tethered to a substrate using dsDNA with biotin and dibenzylcyclooctyne (DBCO) on either end. The remaining streptavidin sites are blocked with poly(ethylene glycol) (PEG)-biotin and PolyT-biotin (see the top-right inset). The dsDNA tether molecule is flexible and allows Brownian motion of the particle in the vicinity of the substrate. The substrate is coated with poly(l-lysine)-*grafted*-poly(ethylene glycol) (PLL-*g*-PEG) and PLL-*g*-PEG-azide, to which DBCO-ssDNA is covalently coupled. Analogues are immobilized on the substrate via DNA hybridization of cortisol–ssDNA conjugates (see the bottom-right inset). The cortisol in solution (blue) and the analogues on the substrate (green) bind reversibly to the antibodies on the particle.

**Figure 2:**
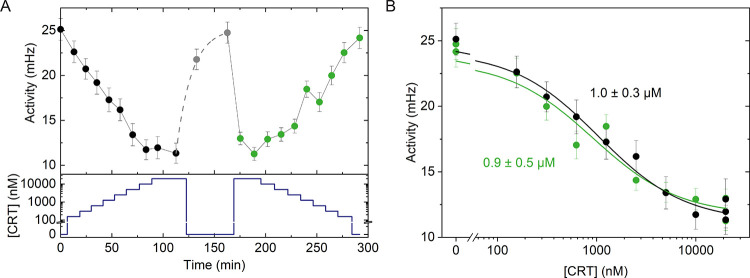
Continuous cortisol monitoring using a BPM competition assay sensor. (A) Continuous cortisol monitoring over 5 h, with the sensor signal in the top panel, and the administered cortisol concentration [CRT] (blue) in the bottom panel. The activity decreases for increasing cortisol concentrations (black) and then shows relaxation upon sensor exposure to a blank solution (gray), followed by a drop in signal in response to a high cortisol concentration, and a steady increase as the cortisol concentration decreases (green). The gray data points are fitted with an exponential decay function with characteristic time of 15 min (dashed gray line). (B) Dose–response curves of the data presented in panel A. The black and green data are similar, demonstrating good stability of the sensor. Data points are fitted with a sigmoidal curve (*y* = *a* + (*b* – *a*)·*x*^*n*^/(EC_50_^*n*^ + *x*^*n*^)) with *n* = 1, resulting in EC_50_ values of 1.0 ± 0.3 and 0.9 ± 0.5 μM, respectively.

This study aims to develop a biosensor for continuous cortisol monitoring in plasma, using a BPM competition immunoassay (Figure 1). In BPM, thousands of particles (Figure S1B) are tracked simultaneously, temporal reversible molecular bonds form between particles and substrate, and these induce observable changes in the mobility of the particles (Figure S1C). The average frequency of bond formation, recorded as the switching activity, relates to the analyte concentration in solution and is used as the signal to monitor the cortisol concentration as a function of time. The particles are functionalized with anti-cortisol antibodies and the substrate with cortisol analogue. In the absence of cortisol, the switching frequency is high as the particle repeatedly binds to and unbinds from the substrate. An increasing cortisol concentration in solution leads to a gradual decrease of the switching frequency of the particles (Figure 2A), as the occupation of antibodies by cortisol from solution lowers the probability that antibodies bind to the cortisol analogues on the substrate.

### Cortisol Monitoring: BPM Sensor Reversibility and Stability

The sensitivity, reversibility, and stability of the sensor are demonstrated by exposing the sensor to series of increasing and decreasing cortisol concentrations (Figure 2). The measured activity represents the average number of switching events per particle per unit of time, with error bars representing the standard error (). The sensor response shows the expected behavior of a BPM competition assay, i.e., the activity signal is inversely proportional to the analyte concentration. In a BPM competition assay, the binding between the antibody and analyte-analogue should be strong enough to cause bound states of the particles that have a sufficiently long lifetime for reliable state detection. However, the binding should not be too strong because long bound-state lifetimes result in low switching activity. In addition, the affinity between the antibody and analyte should be high enough so that low analyte concentrations can be detected. Yet, the affinity should be low enough to permit reversible binding and enable the monitoring of increases as well as decreases of the analyte concentration as a function of time. After comparing different antibodies, we selected one antibody and optimized the densities of the antibody on particles and analyte-analogue on the substrate to achieve single-molecule interactions (see SI 2). This resulted in activity signals in the range of tens of mHz and sensitivity to cortisol concentrations in the range between 100 nM and 10 μM, as shown in Figure 2.

The sensor demonstrates high similarity between consecutively measured dose–response curves, allowing the monitoring of fluctuating cortisol concentrations over a period of 5 h. The measured blank signals at 0, 170, and 300 min are similar within about 10%, demonstrating the stability of the sensor. The observed time of the sensor depends on the concentration step change. In the measurement shown in Figure 2, a decrease in concentration from 30 to 0 μM shows a characteristic time of about 15 min (see the dashed gray curve), while an increase in concentration from 0 to 30 μM gives a response time of about 5 min (visible in the experiment as a rapid signal change between the last gray data point and the first green data point, at *t* = 170 min). The time behavior of the sensor will be further investigated in follow-up research.

### Analysis of State Lifetimes

**Figure 3:**
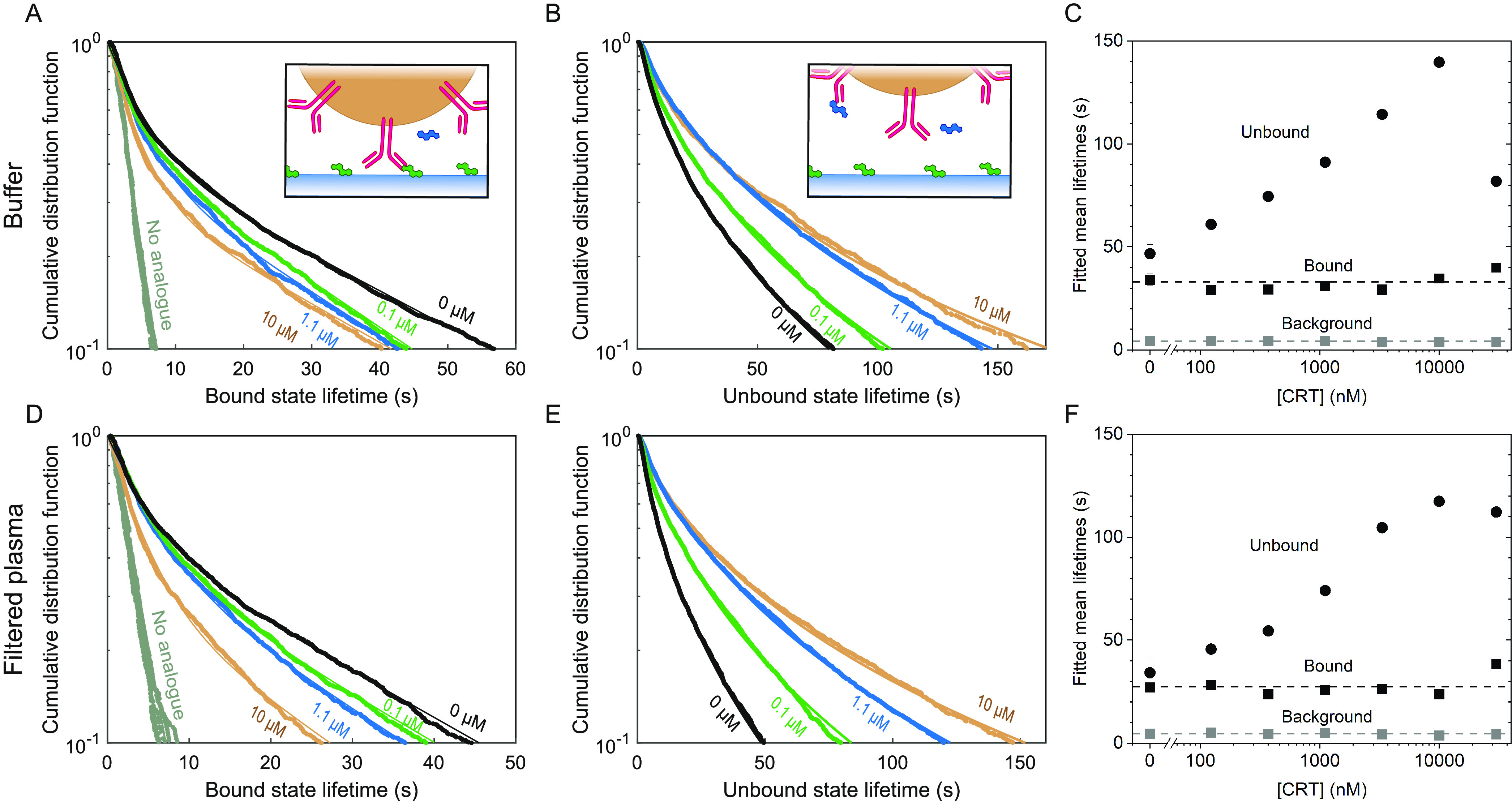
Lifetimes of bound and unbound states in buffer (A–C) and in filtered plasma (D–F). (A) Bound-state lifetime distributions are fitted with double-exponential decay functions with a short and a long mean bound-state lifetime. The short mean lifetimes of 4.2 ± 0.3 s represent the background lifetime, as is also observed for control measurements without analogue molecules on the substrate (gray curve, with a single-exponential behavior). The long mean lifetime of 33.2 ± 3.5 s represents the dissociation of the antibody from the analogue. (B) Unbound-state lifetime distributions are fitted with a multiexponential fit related to the heterogeneity of binder densities on the particles (see Figure S4). The fitted mean unbound-state lifetime increases with increasing cortisol concentration, as antibodies are occupied with cortisol and therefore unable to bind to the analogues on the substrate. (C) Characteristic lifetimes plotted against cortisol concentration [CRT], demonstrating a constant bound-state lifetime (black squares) and unbound-state lifetimes (black circles) depending on the cortisol concentration. The background bound-state lifetime (gray squares) is constant, but the fraction of short-lived bound states depends on the cortisol concentration as visualized in panel (A). The low value of the mean unbound-state lifetime for 30 μM cortisol is an artifact; the lifetime is underestimated because a fraction of the particles remains unbound during the whole measurement. Particles without binding events are excluded from the data sets. (D) Distribution of bound-state lifetimes in 50 kDa filtered blood plasma, fitted with double-exponential decay functions. (E) Distribution of unbound-state lifetimes in filtered blood plasma, fitted with multiexponential decay functions. (F) Characteristic lifetimes plotted against cortisol concentration, demonstrating a constant background (4.5 ± 0.3 s, gray squares), a constant bound-state lifetime (27.3 ± 3.5 s, black squares), and unbound-state lifetimes (black circles) depending on the cortisol concentration. The low unbound-state lifetime for 30 μM cortisol is an artifact (see above).

BPM is a single-molecule technique that allows for investigations of lifetimes of bound and unbound states by analyzing state durations between consecutive switching events. The switching events were determined using the maximum-likelihood multiple-windows change point detection method (MM-CPD).^28^ Particles were classified as bound unless the standard deviation of their *x*–*y* positions exceeded 50 nm over the duration between two switching events. The characteristic duration of antibody–analogue bonds was determined by analyzing distributions of bound-state lifetimes at different conditions, as shown in Figure 3A. First, the bound-state lifetimes in the absence of analogues were analyzed to characterize the background. The cumulative distribution function (CDF) of these states follows a single-exponent decay function (CDF = *e*^–*t*/τ_bg_^) with a mean lifetime of about 4 s (Figure 3A–C). These short-lived states represent the background signal of the experimental system, caused by a combination of nonspecific interactions and misidentified events by the algorithm. The latter relates to the finite MM-CPD window size (set to 0.3–15 s), which was used to identify switching events. Next, the characteristic duration of the single-molecular bonds was determined by analyzing the bound-state lifetimes in the presence of the analogue. To distinguish between background and specific bound-state lifetimes, the cumulative distribution was fitted using a double-exponential function (CDF = *f*_1_·*e*^–*t*/τ_bg_^ + *f*_2_·*e*^–*t*/τ_bound_^, with *f*_1_ + *f*_2_ = 1). The fits reveal two populations of states, with mean lifetimes of ∼4 s and mean lifetimes of around 30 s, respectively, with the fraction of long-lived bound states (*f*_2_) increasing for decreasing cortisol concentrations. The data shows that the mean bound-state lifetimes are independent of the cortisol concentration, which indicates that the sensor forms the same type of bonds over the complete concentration range. By controlling the density of binders on the particle and on the substrate, a sensor was developed that is dominated by single-molecule interactions (analogue-antibody) and not by multivalent bonds between particles and substrate (see Figure S4). The measured bound-state lifetimes correspond to an effective dissociation rate constant of specific bonds on the order of 0.03 s^–1^.

Association processes between the particle and substrate are reflected in the unbound-state lifetimes. Unbound-state lifetimes are defined as the time separation between two bound states and depend on assay conditions such as analogue and antibody density. Particles that remain in the unbound state during the whole measurement duration are not included in the analysis. In the experiments, the number of particles that contribute to the unbound-state lifetimes varied from about 1000 particles for low cortisol concentration to about 500 for the highest cortisol concentration. The cumulative distribution of the unbound-state lifetimes was fitted using a multiexponential decay function, as described by Lubken et al.^26,29^ The multiexponential fitting relates to interparticle heterogeneities of binder densities, in agreement with the fact that measurements on individual particles give single-exponential decay curves; see Figure S4. The obtained mean unbound-state lifetimes increase for higher cortisol concentrations, which is attributed to the higher occupancy of antibody binding sites by cortisol.

Figure 3D–F shows the lifetime analysis of the BPM sensor for cortisol in blood plasma that was filtered with a 50 kDa molecular-weight cutoff. The results in plasma and in the buffer are very similar, for the background, bound-, and unbound-state lifetimes, demonstrating that the biomolecular affinities and nonspecific interactions are hardly affected by filtered plasma.

### Continuous Monitoring of Alternating Cortisol Concentrations in Filtered Blood Plasma

**Figure 4:**
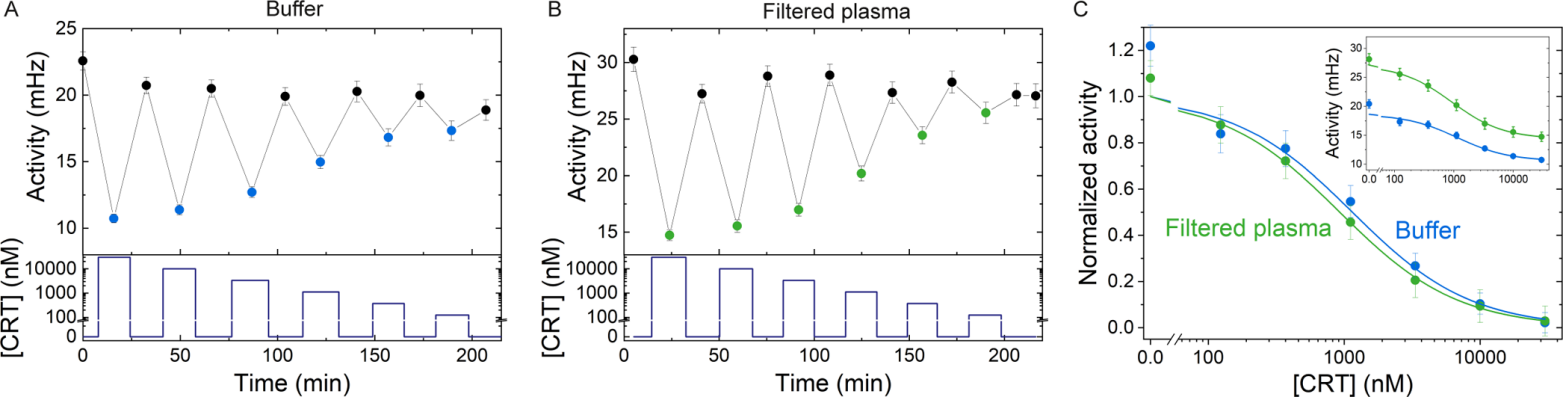
Continuous cortisol monitoring of fluctuating cortisol concentrations in buffer and in 50 kDa filtered blood plasma. (A) Continuous cortisol monitoring over 3.5 h in phosphate-buffered saline (PBS) with 0.5 M NaCl, with switching activity as the sensor signal in the top panel (black), and the administered cortisol concentration [CRT] (dark blue) in the bottom panel. The activity drops for each cortisol sample and returns to the baseline upon supply of a fluid without cortisol. (B) Continuous cortisol monitoring over 3.5 h in 50 kDa filtered blood plasma with 0.5 M NaCl. (C) Dose–response curves of the data depicted in panels A (blue data points) and B (green data points). Black data points are combined in an average value for 0 nM cortisol. The main panel shows the measurement data and fits; for each condition (buffer, plasma), the *y*-axis was rescaled to have normalized sigmoidal fits at zero concentration. The inset shows the measured signals with a sigmoidal fit (*n* = 1), with EC_50_ = 1.1 ± 0.57 × 10^3^ nM (buffer, blue line) and 0.93 ± 0.22 × 10^3^ nM (filtered plasma, green line).

The sensor reversibility and sensor response time were investigated by exposing the sensor to series of large fluctuations of cortisol concentrations, in nanomolar and micromolar ranges (Figure 4). The experiments show that the BPM sensor responds reversibly to increases as well as decreases of cortisol concentrations, with a response time below 15 min, in buffer as well as in filtered blood plasma. The sensors in Figure 4 show blank signals that remain stable within about 10% during the total experiment, as is also seen in Figure 2. However, the absolute value of the signal at zero cortisol concentration differs between individual sensors, as can be seen in Figures 2 and 4. We attribute the differences to variabilities in the surface preparations, causing different areal densities of analogue molecules on the substrate. To compare experiments with different sensors, the data can be plotted with normalized signal values, as shown in Figure 4C. This comparison highlights that experiments in buffer and in blood plasma show sigmoidal curves with strong similarity.

### BPM Measurement with Microdialysis Samples from Plasma

**Figure 5:**
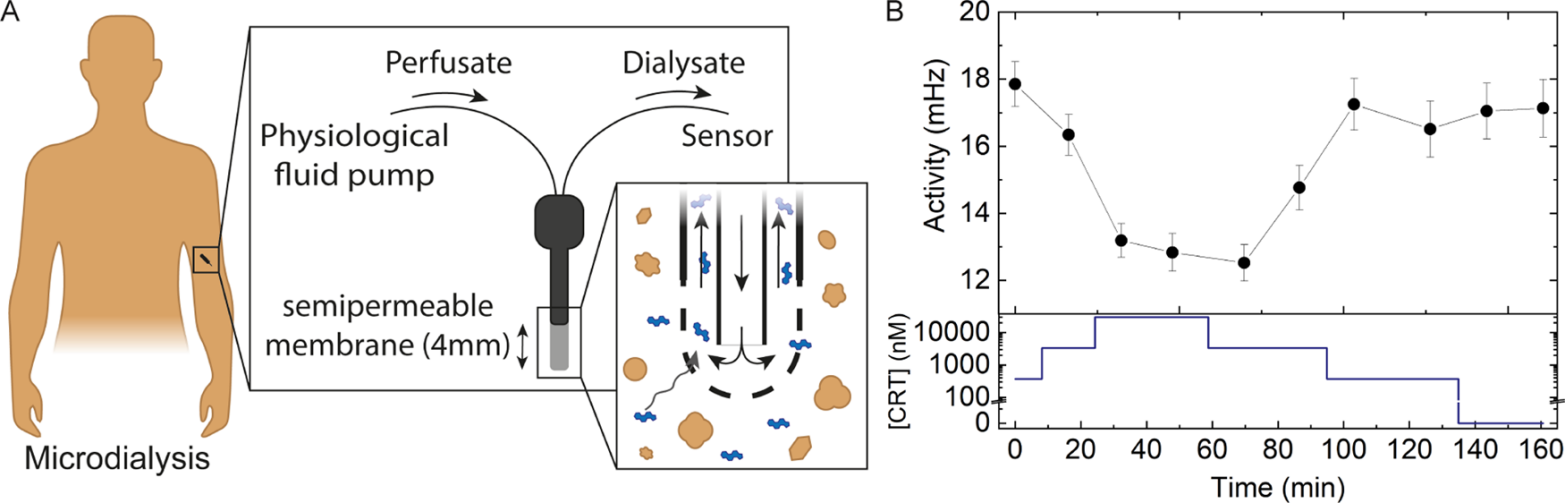
(A) Sketch of envisioned use of a microdialysis probe. The perfusion fluid is flown into the probe, analyte molecules diffuse through the membrane, and the dialysate is transferred to the sensor. (B) Experimental results. Microdialysis samples were taken from blood plasma spiked with cortisol. A probe was used having a 4 mm membrane with a molecular-weight cutoff of 20 kDa. The flow speed was 2 μL per min. The plasma temperature was 37 °C. The bottom panel shows the cortisol concentration in the spiked plasma samples (blue). The top panel shows the BPM sensor signal measured with the dialysate samples.

Microdialysis was selected as a continuous plasma sampling technique because cortisol is a small molecule that can pass through nanofiltration membranes (Table S1) and microdialysis probes are commercially available for use in future clinical applications.^30−32^ Sampling was done from reconstituted lyophilized human blood plasma, spiked with cortisol, and maintained at a temperature of 37 °C. Figure 5A illustrates the principles of a microdialysis probe, with analyte molecules diffusing into a perfusing fluid. The perfusion liquid flowed at a speed of 2 μL per min, the dialysate fluid was collected, and BPM measurements were performed in the dialysate. The BPM sensor signal shows a clear response to increases as well as decreases in cortisol concentration, which demonstrates the feasibility of combining the sampling of cortisol from blood plasma using microdialysis, with measurements on the continuous BPM sensor.

One parameter to characterize microdialysis sampling is the recovery, i.e., the ratio between the concentration of the analyte in the sample and the concentration of the analyte in the dialysate: . Recovery values depend on the analyte, membrane, perfusate, and flow-rate properties. We analyzed different experiments and estimated recoveries in the range between 5 and 35% (Figure S5). Future research will focus on further characterization of recovery and sensing properties for different flows, fluids, probes, and BPM sensor designs.

## Conclusions and Outlook

The goal of this research was to develop a sensor to enable continuous cortisol monitoring in blood plasma and investigate the influence of plasma on the sensing parameters. A BPM sensor was developed with sensitivity in the high nanomolar to low micromolar range, suited for continuous cortisol monitoring over multiple hours in filtered human blood plasma. The reversibility of the sensor was demonstrated by applying alternating cortisol concentrations with increases as well as decreases in cortisol concentration. Reversibility is important for continuous monitoring applications and has been a limitation of cortisol sensors reported in the literature.^20,21,33^

Continuous cortisol monitoring in plasma is useful for mapping stress profiles and inflammatory responses of individual patients. Sampling of blood plasma with a microdialysis probe was demonstrated as an important step toward future real-time patient monitoring. In further research, we will investigate the integration of the BPM sensor with microdialysis sampling, including automated calibrations. BPM as a method for continuous biomolecular sensing^24,27^ and microdialysis as a method for continuous sampling from complex biological fluids^30−32^ are both suited for a wide range of molecules and sample fluids. Therefore, we expect that the combination of BPM and microdialysis will lead to flexible bioanalytical systems for diverse applications in fundamental biological research and patient monitoring.

## Materials and Methods

### Materials

The oligonucleotides used in the study were purchased from IDT. Chemicals used in the study were purchased from Sigma, except if stated otherwise. Custom-made fluid cell stickers (Custom 6 Well Secure Seal) were obtained from Grace Biolabs.

### Preparation of Cortisol–DNA Conjugates

Cortisol 3-CMO-NHS ester (Sigma-Aldrich, H6635) was coupled to ssDNA with a 5′ amine (Amine-5′ -TGG TCT TAC CCC TGC CGC AC-3)′, based on Li et al.,^34^ with the use of HOBt as described by Yan et al.^24^ To obtain cortisol–DNA conjugates, 45 μL of 60 mM cortisol 3-CMO-NHS ester was mixed with 4 μL of 60 mM HOBt (Sigma-Aldrich; 54802), 4 μL of 300 mM 1-ethyl-3-(3-dimethylaminopropyl)carbodiimide (EDC) (Sigma-Aldrich; E6383), and 4 μL of *N*,*N*-diisopropylethylamine (DIPEA) (Sigma-Aldrich; 387649) in dimethylsulfoxide (DMSO). The reaction mixture was incubated at room temperature for 15 min.

Amine-modified DNA was diluted to 10 μM in 3-(*N*-morpholino)propanesulfonic acid (MOPS) buffer (50 mM MOPS (Sigma-Aldrich; M1254) and 0.5 M NaCl, pH 8.0), of which 72 μL was added to the mixture and left to react for 16 h (room temperature, 850 rpm). A fresh reaction mixture of cortisol, HOBt, EDC, and DIPEA was prepared as before, incubated for 15 min, added to the amine–DNA mixture, and left to react for 6 h. The reaction was quenched by adding 25 μL of 500 mM NH_4_OAc (Sigma-Aldrich; A1542).

The reaction mixture containing cortisol–DNA was dissolved in 0.15 mM NaCl in 98% ethanol, stored at −20 °C for 16 h, followed by spinning down at 17,000*g* for 15 min at 4 °C. The pellet was washed a second time (0.15 mM NaCl in 98% ethanol), incubated at −20 °C for 75 min, centrifuged, and washed with 70% ethanol. After incubation at −20 °C for 75 min, it was centrifuged, and the cortisol–DNA was obtained after lyophilization. The cortisol–DNA was dissolved to 25 μM, and the conjugation was verified using gel electrophoresis with a 15% urea gel at 150 V for 90 min.

### Biotinylation of Antibody

The C53 antibody (Thermo Fisher Scientific, Catalog #MA1-83090, 2 mg/mL) was first buffer-exchanged to PBS with Zeba Spin Desalting Columns, 7k MWCO (89882, Thermo Fisher Scientific), according to the manufacturer’s instruction. EZ-Link NHS-PEG4-biotin was dissolved in DMSO at a final concentration of 4 mM. Then, 20-fold molar excess of NHS-PEG4-biotin was added to the antibodies and incubated at room temperature for 1 h. Then, excess NHS-PEG4-biotin was removed by Zeba Spin Desalting Columns, 7k MWCO (89882, Thermo Fisher Scientific), and the biotinylated antibodies were stored in PBS with 0.1% bovine serum albumin (BSA) at a concentration of 1 μM.

### Functionalization of Glass Slides (PLL-*g*-PEG/Azide)

Glass slides (25 × 75 mm, #5, Menzel-Gläser) were cleaned by 30 min of sonication in isopropanol and 30 min of sonication in Milli-Q, after which the glass substrate was dried with a nitrogen stream. The glass substrate was exposed to ozone-plasma for 1 min, followed by attaching a custom-made fluid cell sticker (Grace Biolabs). The fluid cell was filled with 20 μL of 0.45 mg/mL poly(l-lysine)-*grafted*-poly(ethylene glycol) (PLL(20)-*g*[3.5]-PEG(2), SuSoS) and 0.05 mg/mL azide functionalized PLL-*g*-PEG (PLL(15)-g[3.5]-PEG(2)-N3, Nanosoft Biotechnology LLC) as described by Lin et al.^25^ Then, 20 μL of 50 nM of 221 bp dsDNA tether (221 bp dsDNA with biotin on one side and DBCO on the other), diluted in 0.5 M NaCl/PBS, was incubated for approximately 15 h, followed by an incubation with 20 μL of 1 μM ssDNA–DBCO (DBCO-5′-GTG CGG CAG GGG TAA GAC CA-3′) for at least 48 h (RT, up to several months).

### Functionalization of Particles

Streptavidin-coated magnetic particles (10 mg/mL, Dynabeads MyOne Streptavidin C1, 65001, Thermo Scientific) were incubated for 30 min with 2 μL of 125 nM biotinylated cortisol antibodies with a total volume of 4 μL, on a rotating fin. Subsequently, 5 μL of 2 μM PolyT-biotin (Biotin-5′-TTT TTT TTT TTT TTT T-3′) was added and incubated for 30 min on a rotating fin. The particle mixture was washed with 500 μL of 0.05% Tween20 in PBS using magnetic separation, and the particles were reconstituted in 300 μL of 0.5 M NaCl/PBS. Finally, the particle mixture was sonicated with 10 pulses at 70% with a 0.5 duty cycle (Hielscher, Ultrasound Technology).

### Plasma and Cortisol Preparation

Human blood plasma (Sigma P9523-5 mL) was reconstituted using 5 mL of Milli-Q. The 50 kDa filtered plasma was prepared by mixing it with 5 M NaCl/PBS to reach 0.5 M NaCl/plasma, which was filtered using a 50 kDa molecular-weight cutoff centrifugal filter (UFC905008, Millipore) according to the supplier’s protocol (total centrifugation time of 20 min).

Cortisol stock was prepared by dissolving 1 mg/mL in methanol (technical grade) and diluted further in either 0.5 M NaCl/PBS, 50 kDa filtered plasma with 0.5 M NaCl, or full plasma, with concentrations ranging from 30 μM down to 123 nM.

### Sensor Assembly and Cortisol Detection

On the day of use, 250 μL of functionalized particles was injected (Harvard pump 11 Elite, 40 μL/min withdrawal speed) into the fluid cell (Grace BioLabs). Particles were incubated for 5 min to allow particles to sediment to the substrate and attach to the DNA tethers. Thereafter, the slide was reversed to allow untethered particles to sediment away from the functionalized surface. Second, 400 μL of 100 μM of 1 kDa mPEG-biotin (PG1-BN-1k, Nanocs) was added, which was incubated for 30 min. During incubation, the tethered particles were measured to determine the background signal. Activation of the system was done by adding 200 μL of 250–500 pM cortisol–DNA (analogue) and incubated for 20 min. Excess analogue was removed with 200 μL of 0.5 M NaCl/PBS, after which the media containing varying cortisol concentrations were added, with 200 μL for each sample. Particle motion was recorded for 5 (Figure 2) or 10 min, in the absence of flow. Reported steps in the bottom panels of Figure 2, 4, and 5 represent start of sample injection into the cartridge. Time values of the datapoints in the top panels are positioned in the middle of the total duration of fluid injection and particle motion recording.

### Microdialysis

A microdialysis probe (CMA/20, 20 kDa MWCO, 4 mm) was placed in a container with sample media (PBS or undiluted blood plasma), heated to 37 °C. A syringe pump (Harvard Pico Elite) was used to infuse at the rate of 2 μL/min. A 10 mL syringe (airtight) was filled with PBS, and each time, the pump was turned on to inject 30 μL when collecting samples from the media. Per injection, 27 μL of collected sample was combined with 3 μL of 5 M NaCl/PBS, pipetted into the BPM sensor fluid cell, and recorded for 10 min.

Based on switching activity values obtained from calibration points (buffer and 30 μM cortisol), the a- and b-values of the sigmoidal curve () were fitted by fixing EC_50_ = 928 nM and *n* = 1. The cortisol concentration was calculated based on the measured switching activity, followed by correcting for sample dilution. Recovery was calculated by comparing the detected dialysate cortisol concentration with the sample concentration () using linear fitting through zero.

### Image Recording and Data Analysis

Tethered particles were tracked before, during, and after analogue binding, and after each concentration change, on a Leica Microscope (DMI5000 M with a CTR6000 light source), at a total magnification of 10× using a high-speed FLIR CMOS camera (Point Grey Research Grashopper3 GS3-U3-23S6M, 1920 × 1200, pixel format: 8 raw, Gain 10). The particle motion in a field of view of 1129 × 706 μm^2^ was recorded for 5–10 min at the frame rate of 30 Hz with 5 ms exposure time under dark-field illumination conditions. The localization of particles was done using phasor-based localization.^35^ The analysis of particle motion and detection of switching events were done using the maximum-likelihood multiple-windows change point detection method (MM-CPD)^28^ and the method described in an earlier publication.^23^

## Supporting Information Available

Principle of biosensing by particle mobility, analogue synthesis scheme, antibody selection, assay optimization, sensor specificity, state lifetimes of individual particles, and microdialysis recovery (PDF)

## Abbreviations

BPM: biosensing by particle mobility
CRT: cortisol
MWCO: molecular-weight cutoff
SE: standard error
MM-CPD: maximum-likelihood multiple-windows change point detection
CDF: cumulative distribution function

## References

[ref1] Chrousos G. P. Stress and disorders of the stress system. Nat. Rev. Endocrinol. 2009, 5, 374–381. 10.1038/nrendo.2009.106. 19488073

[ref2] Matyszak M. K.; Citterio S.; Rescigno M.; Ricciardi-Castagnoli P. Differential effects of corticosteroids during different stages of dendritic cell maturation. Eur. J. Immunol. 2000, 30, 1233–1242. 10.1002/(SICI)1521-4141(200004)30:4<1233::AID-IMMU1233>3.0.CO;2-F. 10760813

[ref3] Meyer E. J.; Nenke M. A.; Lewis J. G.; Torpy D. J. Corticosteroid-binding globulin: acute and chronic inflammation. Expert Rev. Endocrinol. Metab. 2017, 12, 241–251. 10.1080/17446651.2017.1332991. 30058887

[ref4] Kim Y. J.; Kim J. H.; Hong A. R.; Park K. S.; Kim S. W.; Shin C. S.; Kim S. Y. Stimulated Salivary Cortisol as a Noninvasive Diagnostic Tool for Adrenal Insufficiency. Endocrinol. Metab. 2020, 35, 628–635. 10.3803/EnM.2020.707. PMC7520577 32981305

[ref5] El-Farhan N.; Rees D. A.; Evans C. Measuring cortisol in serum, urine and saliva—are our assays good enough?. Ann. Clin. Biochem. 2017, 54, 308–322. 10.1177/0004563216687335. 28068807

[ref6] Azmi N. A. S. M.; Juliana N.; Azmani S.; Effendy N. M.; Abu I. F.; Teng N. I. M. F.; Das S. Cortisol on circadian rhythm and its effect on cardiovascular system. Int. J. Environ. Res. Public Health 2021, 18, 1–15. 10.3390/ijerph18020676 PMC7830980 33466883

[ref7] Venugopal M.; Arya S. K.; Chornokur G.; Bhansali S. A realtime and continuous assessment of cortisol in ISF using electrochemical impedance spectroscopy. Sens. Actuators, A 2011, 172, 154–160. 10.1016/j.sna.2011.04.028. PMC3234992 22163154

[ref8] Nielsen N. R.; Kristensen T. S.; Strandberg-Larsen K.; Zhang Z. F.; Schnohr P.; Grønbæk M. Perceived stress and risk of colorectal cancer in men and women: A prospective cohort study. J. Intern. Med. 2008, 263, 192–202. 18226096 10.1111/j.1365-2796.2007.01826.x

[ref9] Chrousos G. P.; Gold P. W. The Concepts of Stress and Stress System Disorders: Overview of Physical and Behavioral Homeostasis. J. Am. Med. Assoc. 1992, 267, 1244–1252. 10.1001/jama.1992.03480090092034. 1538563

[ref10] McEwen B. S. Cortisol, Cushing’s Syndrome, and a Shrinking Brain—New Evidence for Reversibility. J. Clin. Endocrinol. Metab. 2002, 87, 1947–1948. 10.1210/jc.87.5.1947. 11994322

[ref11] Pearlmutter P.; DeRose G.; Samson C.; Linehan N.; Cen Y.; Begdache L.; Won D.; Koh A. Sweat and saliva cortisol response to stress and nutrition factors. Sci. Rep. 2020, 10, 1220 10.1038/s41598-020-75871-3. 33149196 PMC7643128

[ref12] Dalirirad S.; Han D.; Steckl A. J. Aptamer-Based Lateral Flow Biosensor for Rapid Detection of Salivary Cortisol. ACS Omega 2020, 5, 32890–32898. 10.1021/acsomega.0c03223. 33403250 PMC7774066

[ref13] Sekar M.; Pandiaraj M.; Bhansali S.; Ponpandian N.; Viswanathan C. Carbon fiber based electrochemical sensor for sweat cortisol measurement. Sci. Rep. 2019, 9, 403 10.1038/s41598-018-37243-w. 30674991 PMC6344552

[ref14] Darwish I. A. Immunoassay Methods and their Applications in Pharmaceutical Analysis: Basic Methodology and Recent Advances. Int. J. Biomed. Sci. 2006, 2, 217–235. 23674985 PMC3614608

[ref15] Sheibani S.; Capua L.; Kamaei S.; Akbari S. S. A.; Zhang J.; Guerin H.; Ionescu A. M. Extended gate field-effect-transistor for sensing cortisol stress hormone. Commun. Mater. 2021, 2, 1–10. 10.1038/s43246-020-00114-x. PMC7815575 33506228

[ref16] Nandhakumar P.; Haque A. M. J.; Lee N. S.; Yoon Y. H.; Yang H. Washing-Free Displacement Immunosensor for Cortisol in Human Serum Containing Numerous Interfering Species. Anal. Chem. 2018, 90, 10982–10989. 10.1021/acs.analchem.8b02590. 30148606

[ref17] Torrente-Rodríguez R. M.; Tu J.; Yang Y.; Min J.; Wang M.; Song Y.; Yu Y.; Xu C.; Ye C.; IsHak W. W.; Gao W. Investigation of Cortisol Dynamics in Human Sweat Using a Graphene-Based Wireless mHealth System. Matter 2020, 2, 921–937. 10.1016/j.matt.2020.01.021. 32266329 PMC7138219

[ref18] Parlak O. Portable and Wearable Real-Time Stress Monitoring: A Critical Review. In Sensors and Actuators Reports; Elsevier, 2021; Vol. 3, p 100036.

[ref19] Parlak O.; Keene S. T.; Marais A.; Curto V. F.; Salleo A. Molecularly selective nanoporous membrane-based wearable organic electrochemical device for noninvasive cortisol sensing. Sci. Adv. 2018, 4, 1–10. 10.1126/sciadv.aar2904. PMC6054510 30035216

[ref20] Tang W.; Yin L.; Sempionatto J. R.; Moon J. M.; Teymourian H.; Wang J. Touch-Based Stressless Cortisol Sensing. Adv. Mater. 2021, 33, 2008465 10.1002/adma.202008465. 33786887

[ref21] An J. E.; Kim K. H.; Park S. J.; Seo S. E.; Kim J.; Ha S.; Bae J.; Kwon O. S. Wearable Cortisol Aptasensor for Simple and Rapid Real-Time Monitoring. ACS Sens. 2022, 7, 99–108. 10.1021/acssensors.1c01734. 34995062

[ref22] Wang B.; Zhao C.; Wang Z.; Yang K. A.; Cheng X.; Liu W.; Yu W.; Lin S.; Zhao Y.; Cheung K. M.; Lin H.; Hojaiji H.; Weiss P. S.; Stojanović M. N.; Tomiyama A. J.; Andrews A. M.; Emaminejad S. Wearable aptamer-field-effect transistor sensing system for noninvasive cortisol monitoring. Sci. Adv. 2022, 8, 1–16. 10.1126/sciadv.abk0967. PMC8730602 34985954

[ref23] Visser E. W. A.; Yan J.; Van IJzendoorn L. J.; Prins M. W. J. Continuous biomarker monitoring by particle mobility sensing with single molecule resolution. Nat. Commun. 2018, 9, 2541 10.1038/s41467-018-04802-8. 29959314 PMC6026194

[ref24] Yan J.; Van Smeden L.; Merkx M.; Zijlstra P.; Prins M. W. J. Continuous Small-Molecule Monitoring with a Digital Single-Particle Switch. ACS Sens. 2020, 5, 1168–1176. 10.1021/acssensors.0c00220. 32189498 PMC8177406

[ref25] Lin Y. T.; Vermaas R.; Yan J.; De Jong A. M.; Prins M. W. J. Click-Coupling to Electrostatically Grafted Polymers Greatly Improves the Stability of a Continuous Monitoring Sensor with Single-Molecule Resolution. ACS Sens. 2021, 6, 1980–1986. 10.1021/acssensors.1c00564. 33985333 PMC8165697

[ref26] Lubken R. M.; De Jong A. M.; Prins M. W. J. Multiplexed Continuous Biosensing by Single-Molecule Encoded Nanoswitches. Nano Lett. 2020, 20, 2296–2302. 10.1021/acs.nanolett.9b04561. 32091908 PMC7252944

[ref27] Lubken R. M.; Bergkamp M. H.; de Jong A. M.; Prins M. W. J. Sensing Methodology for the Rapid Monitoring of Biomolecules at Low Concentrations over Long Time Spans. ACS Sens. 2021, 6, 1c01991 10.1021/acssensors.1c01991. 34854303 PMC8715529

[ref28] Bergkamp M. H.; Van IJzendoorn L. J.; Prins M. W. J. Real-Time detection of state transitions in stochastic signals from biological systems. ACS Omega 2021, 6, 17726–17733. 10.1021/acsomega.1c02498. 34278158 PMC8280633

[ref29] Lubken R. M.; de Jong A. M.; Prins M. W. J. How Reactivity Variability of Biofunctionalized Particles Is Determined by Superpositional Heterogeneities. ACS Nano 2021, 15, 1331–1341. 10.1021/acsnano.0c08578. 33395272 PMC7844819

[ref30] Bhake R.; Russell G. M.; Kershaw Y.; Stevens K.; Zaccardi F.; Warburton V. E. C.; Linthorst A. C. E.; Lightman S. L. Continuous free cortisol profiles in healthy men: Validation of microdialysis method. J. Clin. Endocrinol. Metab. 2020, 105, E1749–E1761. 10.1210/clinem/dgz002. 31529059

[ref31] Joukhadar C.; Uller M. Microdialysis Current Applications in Clinical Pharmacokinetic Studies and its Potential Role in the Future. Clin. Pharmacokinet. 2005, 44, 895–913. 10.2165/00003088-200544090-00002. 16122279

[ref32] Plock N.; Kloft C. Microdialysis-Theoretical background and recent implementation in applied life-sciences. Eur. J. Pharm. Sci. 2005, 25, 1–24. 10.1016/j.ejps.2005.01.017. 15854796

[ref33] Safarian S. M.; Kusov P. A.; Kosolobov S. S.; Borzenkova O. V.; Khakimov A. V.; Kotelevtsev Y. V.; Drachev V. P. Surface-specific washing-free immunosensor for time-resolved cortisol monitoring. Talanta 2021, 225, 122070 10.1016/j.talanta.2020.122070. 33592788

[ref34] Li Y.; Gabriele E.; Samain F.; Favalli N.; Sladojevich F.; Scheuermann J.; Neri D. Optimized Reaction Conditions for Amide Bond Formation in DNA-Encoded Combinatorial Libraries. ACS Comb. Sci. 2016, 18, 438–443. 10.1021/acscombsci.6b00058. 27314981 PMC5515351

[ref35] Martens K. J. A.; Bader A. N.; Baas S.; Rieger B.; Hohlbein J. Phasor based single-molecule localization microscopy in 3D (pSMLM-3D): An algorithm for MHz localization rates using standard CPUs. J. Chem. Phys. 2018, 148, 123311 10.1063/1.5005899. 29604874

