# Supplementary material for: Reversible Immunosensor for the Continuous Monitoring of Cortisol in Blood Plasma Sampled with Microdialysis
Source: ACS Sens. 2022 Oct 18;7(10):3041–8. doi: 10.1021/acssensors.2c01358 (PMC9623578; doi:10.1021/acssensors.2c01358)
Supplement: Supplementary file 1 — se2c01358_si_001.pdf [file se2c01358_si_001.pdf]

Reversible Immunosensor for the Continuous Monitoring of Cortisol in Blood Plasma Sampled with Microdialysis

Laura van Smeden[1,3], Annet Saris[1], Khulan Sergelen[1,3], Arthur M. de Jong[2,3], Junhong Yan[4⊥], Menno W. J. Prins[1,2,3,4*⊥]

[1]Department of Biomedical Engineering, Eindhoven University of Technology, 5600 MB Eindhoven, The Netherlands.
[2]Department of Applied Physics, Eindhoven University of Technology, 5600 MB Eindhoven, The Netherlands.
[3]Institute for Complex Molecular Systems (ICMS), Eindhoven University of Technology, 5600 MB Eindhoven, The Netherlands.
[4]Helia Biomonitoring, De Lismortel 31, 5612 AR Eindhoven, The Netherlands.

Supporting Information

CONTENTS

SI 1: Principle of Biosensing by Particle Mobility 2

SI 2: Antibody selection and assay optimization for single-molecule binding 3

SI 3: Specific cortisol detection 5

SI 4: State lifetimes of individual particles 6

SI 5: Microdialysis recovery 7

Supporting methods 9

References 10

SI 1: PRINCIPLE OF BIOSENSING BY PARTICLE MOBILITY

Biosensing by Particle Mobility (BPM) is based on tracking thousands of particles (Ø 1µm; Figure S1) using dark field microscopy with 10× magnification. Each particle is tracked in real-time using phasor-based localization[1], and transitions in the particle mobility are extracted to determine the switching events between the two states (bound and unbound) using change point detection, as described by Bergkamp et al.[2].

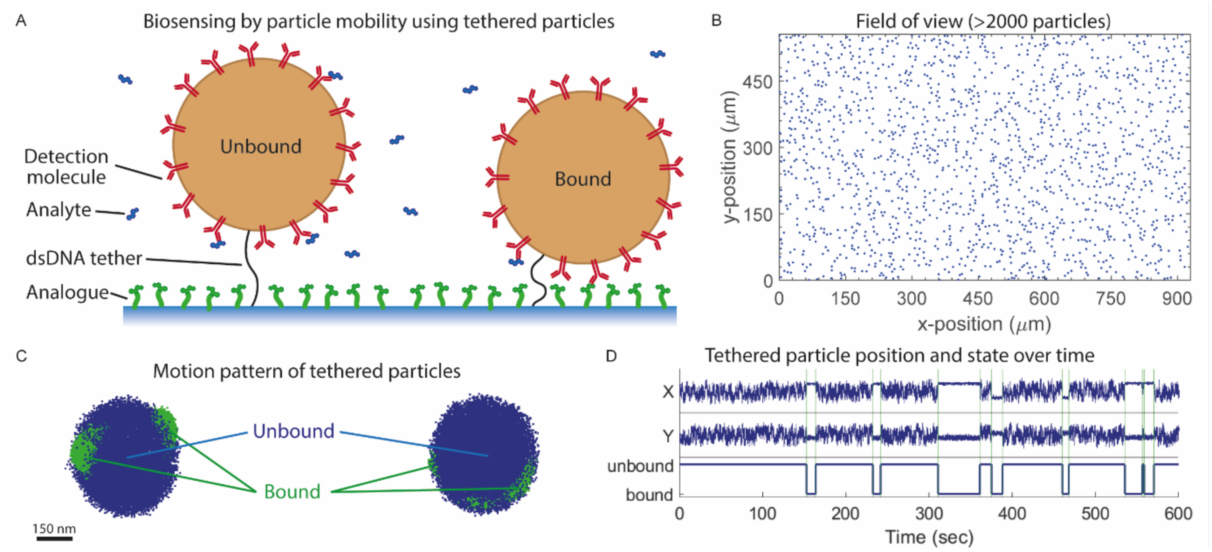

Figure S1: Biosensing by particle mobility. A) Particles are functionalized with antibodies as detection molecules and tethered to a substrate using dsDNA. The substrate is functionalized with analogue to which the detection molecules can bind to induce a bound state. The bond is affinity-based and therefore reversible, allowing the particle to return to the unbound state. B) Thousands of particles are localized and tracked simultaneously in a field of view. C) Tethered particles show a circular motion pattern in their unbound state (blue), and when the particle binds to the substrate, the mobility decreases, leading to smaller motion patterns (green). D) Particle mobility over time indicated by the x- and y-position with green lines indicating (un)binding events. The bottom line shows the identified state at that moment in time. A competition assay shows minimal binding for high analyte concentration and frequent binding for low analyte concentrations as presented here.

The bound state is induced by the stochastic probability of binding of antibody on the particle to analogue on the substrate. The analogue was synthesized by conjugating cortisol to ssDNA, via the reaction scheme of Figure S2. This approach of analogue synthesis was demonstrated by Yan et al. [3].

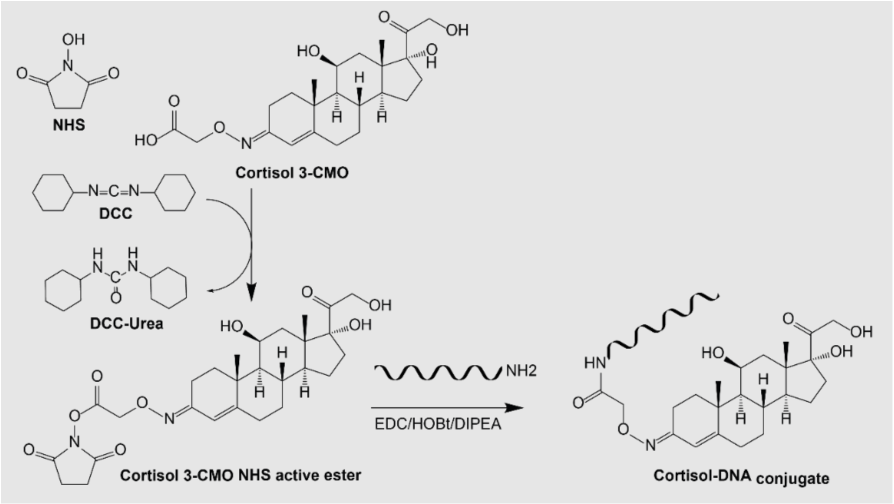

Figure S2: Synthesis of cortisol-DNA conjugate via NHS activation and coupling to ssDNA-amine. Details are provided in the methods section.

SI 2: ANTIBODY SELECTION AND ASSAY OPTIMIZATION FOR SINGLE-MOLECULE BINDING

Three anti-cortisol antibodies were compared in this study, having as immunogens cortisol-3-CMO-BSA, cortisol conjugated to BSA, and synthetic corticosterone conjugated to BSA, corresponding with F4P1A3, C42, and C53, respectively. Screening (Figure S3) was done using antibodies physisorbed on the substrate and cortisol analogue coupled to the particles (see Supporting Methods).

Figure S3 demonstrates that of the screened antibodies, C53 has the most potential. It shows a clear increase in switching activity upon increase of cortisol analogue and the highest value of switching activity (panel A). Decreasing the C53 antibody concentration from 100 µg/ml to 50 and 25 µg/ml resulted in a lower switching activity (panel B) and an increase in the fraction of long-lived bound states (panel C). The characteristic lifetimes of the long-lived bound states are comparable for both concentrations (~15 s), indicating that the underlying interactions are equal. These are attributed to single-molecule antibody-analogue bonds. The bond lifetimes are short and allow for measurements with a high switching activity.

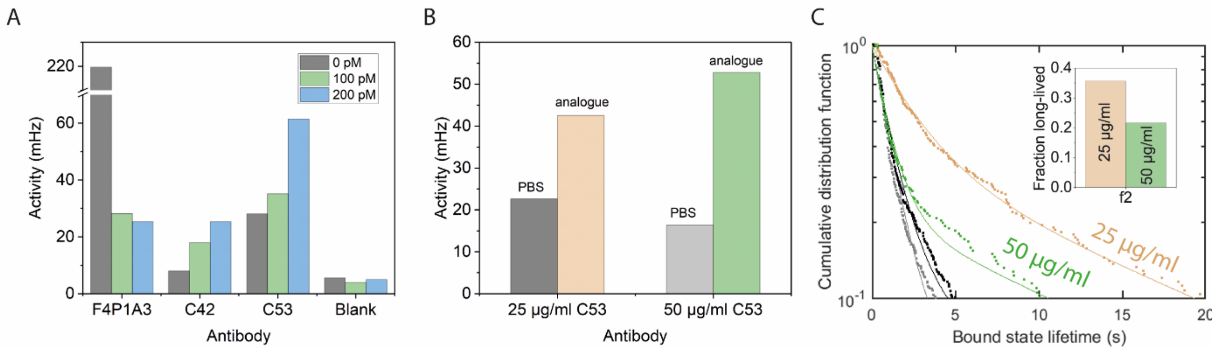

Figure S3: Screening of antibodies and concentrations. A) Measured switching activity in the absence (0 pM) and presence of analogue (100 and 200 pM), for 100 µg/ml physisorbed antibodies on a substrate compared with blank (no antibody). B) Comparison of switching activity for 25 and 50 µg/ml physisorbed C53 antibody, in the absence of analogue (PBS) and with 250 pM analogue. C) Bound-state lifetimes of data from panel B, fitted with a double exponential fit. Inset shows the fraction of long-lived states with 250 pM analogue.

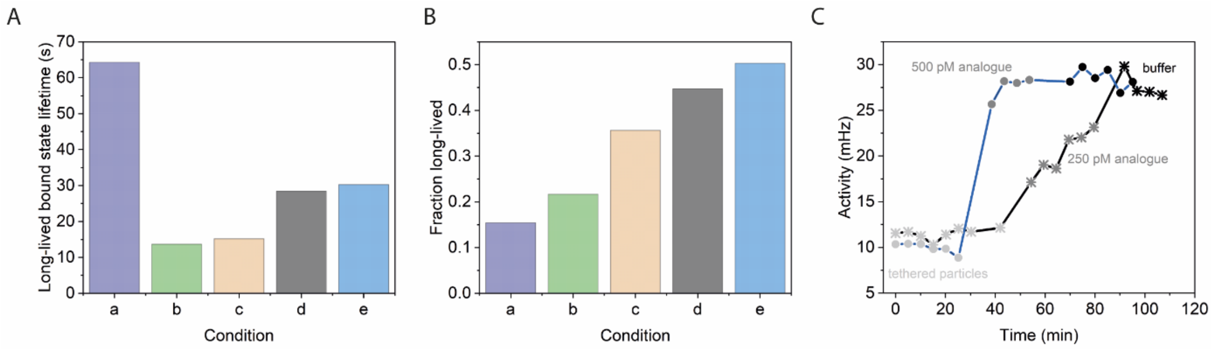

Figure S4: Optimization steps for single-molecule binding. A) The long-lived bound-state lifetime for the conditions a) substrate: 100 µg/ml C53 & particle: 200 pM analogue, b) substrate: 50 µg/ml C53 & particle: 250 pM analogue, c) substrate: 25 µg/ml C53 & particle: 250 pM analogue, d) substrate: 250 pM analogue & particle: 125 nM C53-biotin, e) substrate: 500 pM analogue & particle: 125 nM C53-biotin. B) The fraction of long-lived states for the conditions as described in panel A. C) Comparison of analogue incorporations over time. The black line indicates condition (d) with 250 pM analogue (star-shaped markers). The blue line indicates condition (e) with 500 pM analogue (circle-shaped markers). Light gray markers indicate the signal of tethered particles before analogue addition and gray markers the signal upon analogue incubation resulting in an increase of reversible particle-surface interactions. Black markers indicate the signal after exposing the sensor to a blank buffer solution which shows that the signal after the functionalization step remains stable.

Based on the screening results (Figure S3), the assay was further developed using C53 antibodies. Figure S4 shows the mean bound-state lifetimes for a variety of assay conditions (panel A). This comparison includes assays with antibodies physisorbed on the substrate (conditions a, b, c) and assays with analogue hybridized to the substrate using PLL-g-PEG/azide conjugated with ssDNA-DBCO (conditions d, e). The PLL-g-PEG systems gives a large fraction of states with bound-state lifetimes of about 30 s, with very little dependence on analogue concentration (250 pM, 500 pM). This indicates that the BPM sensor is tuned into a regime where the binding is dominated by single-molecular bonds. Panel C shows the time behavior of the incorporation of analogue into the sensor. Both analogue concentrations give the same end value of switching activity, but the incorporation goes faster with the higher analogue concentration (500 pM).

The results in Figures S3 and S4 show how a BPM sensor can be developed with bound states dominated by single-molecular bonds. The bound-state lifetimes are a result of the binding strength between individual antibody and analogue molecules, but are also influenced by physicochemical forces between particle and substrate, such as charge-based interactions, steric, van der Waals, etc. Here, the applied blocking strategies play an important role. The BPM sensor is developed to have minimal particle binding in absence of antibody or analogue. Therefore, the net physicochemical forces between particle and substrate are in principle repulsive. The strength of the repulsive force depends on the conditions of particle, substrate, and solution. In the study of Figures S3 and S4, the measured bound-state lifetimes were slightly lower in the physisorbed system (~15 s) compared to the PLL-g-PEG system (~30 s), indicating that the repulsive force between particle and substrate may have been somewhat higher in the physisorbed system.

The effective association rate between particle and substrate depends on the densities of analogue and antibody molecules on the substrate and particle, but also on the diffusivity of the particle and the average distance between particle and substrate. The EC_{50} (half maximum effective concentration) measured in the dose-response curves reflects the interaction strength between antibodies in the sensor and cortisol free in solution (Kd). However, the EC_{50} value can also depend on the speed of the assay (is equilibrium reached or not) and on possible saturations of the sensor signal (is the maximum sensor signal limiting or not). In the experiments of this paper, EC_{50} values were observed of about 1 μM. In follow-up research we will further investigate the dependencies of EC_{50} on experimental conditions. The sigmoidal fits of the dose-response curves had slopes with n=1. This indicates an absence of cooperativity, in agreement with particle-surface interactions dominated by single-molecule antibody-analogue bonds.

The response time of the sensor was observed to be ~5 min (see low to high concentration in Figure 2) and ~15 min (see high to low concentration in Figure 2). The response time is influenced by the applied measurement time (5 min), by transport processes in the cartridge (advection, diffusion) and by molecular binding processes (association, dissociation). The mean bound-state lifetime of particles bound to the substrate via antibody-analogue bonds is ~30 s (see Figure 3). Therefore, the measurement time and transport processes are expected to significantly contribute to the observed response time. These topics will be investigated in follow-up research.

SI 3: SPECIFIC CORTISOL DETECTION

The specificity of the BPM sensor for measuring cortisol is demonstrated in Figure S5, where the sensor was exposed to cortisol as well as danazol, a steroid hormone similar to cortisol. The results show that danazol does not bind to the antibodies, as the activity does not decrease compared to detection in buffer.

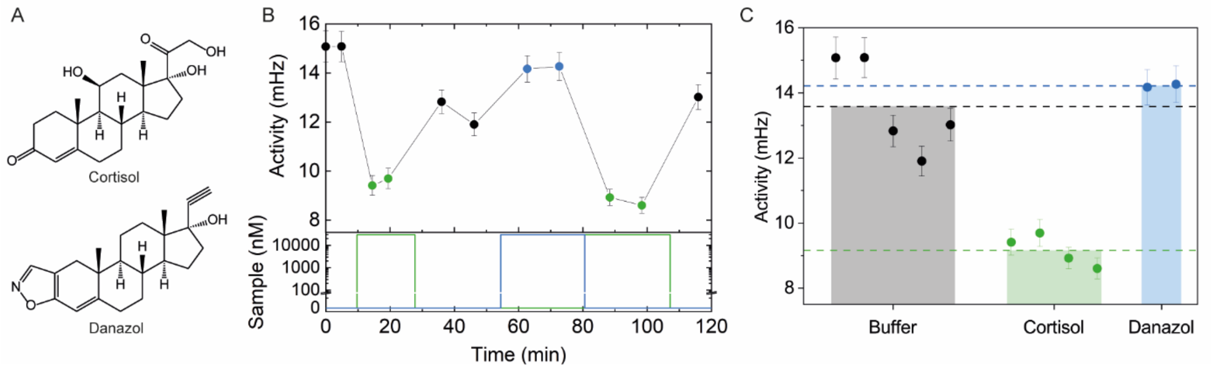

Figure S5: Specificity of the cortisol sensor. A) Chemical structure of cortisol (top) and danazol (bottom). B) Continuous cortisol monitoring over 2 hours, with the sensor signal in the top panel, and the administered sample (cortisol in green, danazol in blue) concentration in the bottom panel. Specificity for cortisol demonstrated by comparing signal for buffer (black), 30 μM cortisol (green), and 30 μM danazol (blue). C) Scatterplot of categorized data obtained from panel B. Bars and dashed lines indicate the average activity per category.

SI 4: STATE LIFETIMES OF INDIVIDUAL PARTICLES

The lifetimes of bound and unbound states of particles give information about the nature of the binding between particle and substrate. The lifetimes of bound and unbound states were analyzed on the single-particle level (Figure S6) and for all particles combined (ensemble level) (Figure 3). The unbound-state lifetimes are defined as the duration between two bound states. For individual particles, the cumulative distribution functions of unbound-state lifetimes show a single-exponential behavior, with varying unbound-state lifetimes (Figure S6A). The distribution of those lifetimes has the shape of a log-normal distribution (μ=3.1, σ=0.7), meaning that the ensemble of unbound-state lifetimes is expected to follow a multi-exponential curve[4,5].

The mean unbound-state lifetimes fitted with multi-exponential curves are shown in Figure 3B and 3E. As indicated in the text, particles remaining in the unbound state during the total measurement, were excluded from the analysis. This gives an underestimation of the lifetime, particularly in cases of low switching activity, as observed for 30 μM cortisol (~2200 observed states based on ~500 particles). This means that when the particles show a low switching activity, the unbound-state lifetimes are dominated by background (non-specific interactions and data processing artefacts). This is also seen for particles in the absence of analogue, where the mean unbound-state lifetime is 73±3 s.

The CDF of the bound-state lifetimes on the single-particle level is shown in panel C. The figure shows a single-exponential behavior and the distribution of the obtained characteristic bound-state lifetimes is also single-exponential (μ=18). Lifetime distributions are fitted with a double-exponential (see Figure 3A and 3D); the long-lived states (~30 s) are attributed to specific interactions, the short-lived states (~3 s) to background. The bound-state lifetimes assigned to background are also observed in assays without analogue on the substrate and are therefore assigned to non-specific interactions and/or data processing artefacts.

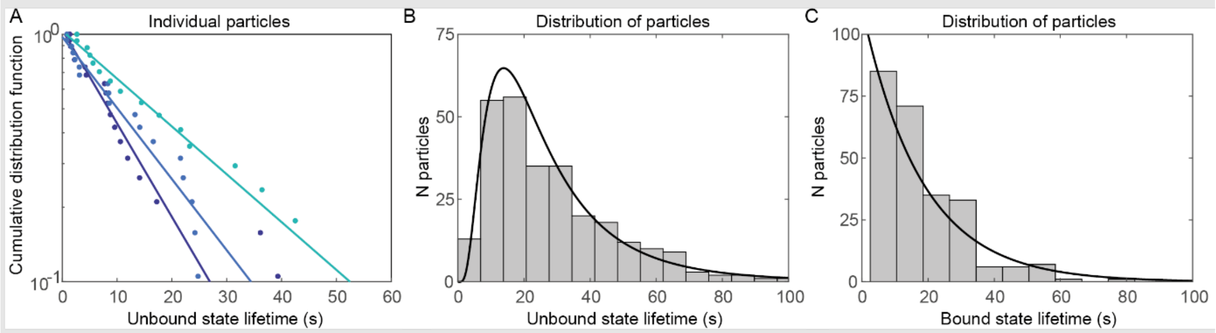

Figure S6: Single-particle state lifetime analysis and the distribution of state lifetimes. A) Unbound-state lifetimes of three individual particles, fitted with a single exponent. B) Distribution of characteristic unbound-state lifetimes of particles (obtained via fitting of individual particle unbound-state lifetimes, as in panel A) plotted in a histogram, with a log-normal distribution fit (μ=3.1, σ=0.7). C) Distribution of characteristic bound-state lifetimes of particles plotted in a histogram with a single-exponential distribution (μ=18).

SI 5: MICRODIALYSIS RECOVERY

Microdialysis was selected as method to continuously sample from human blood plasma. As proof of concept, samples were taken by microdialysis, and these were measured on a cortisol BPM sensor. The cortisol recovery was determined by comparing the sensor signal to a calibration curve. The dose-response curve was calibrated using control measurements (0 and 30 µM cortisol, Figure S7) to obtain the range of sensitivity and using a sigmoidal curve with a fixed EC_{50} of 928 nM obtained from an earlier experiment (see filtered plasma Figure 4). The error of the EC_{50} value and calibration points were neglected; only the error of the signal (in the form of the activity) was used when translating the signal towards concentrations (see Methods).

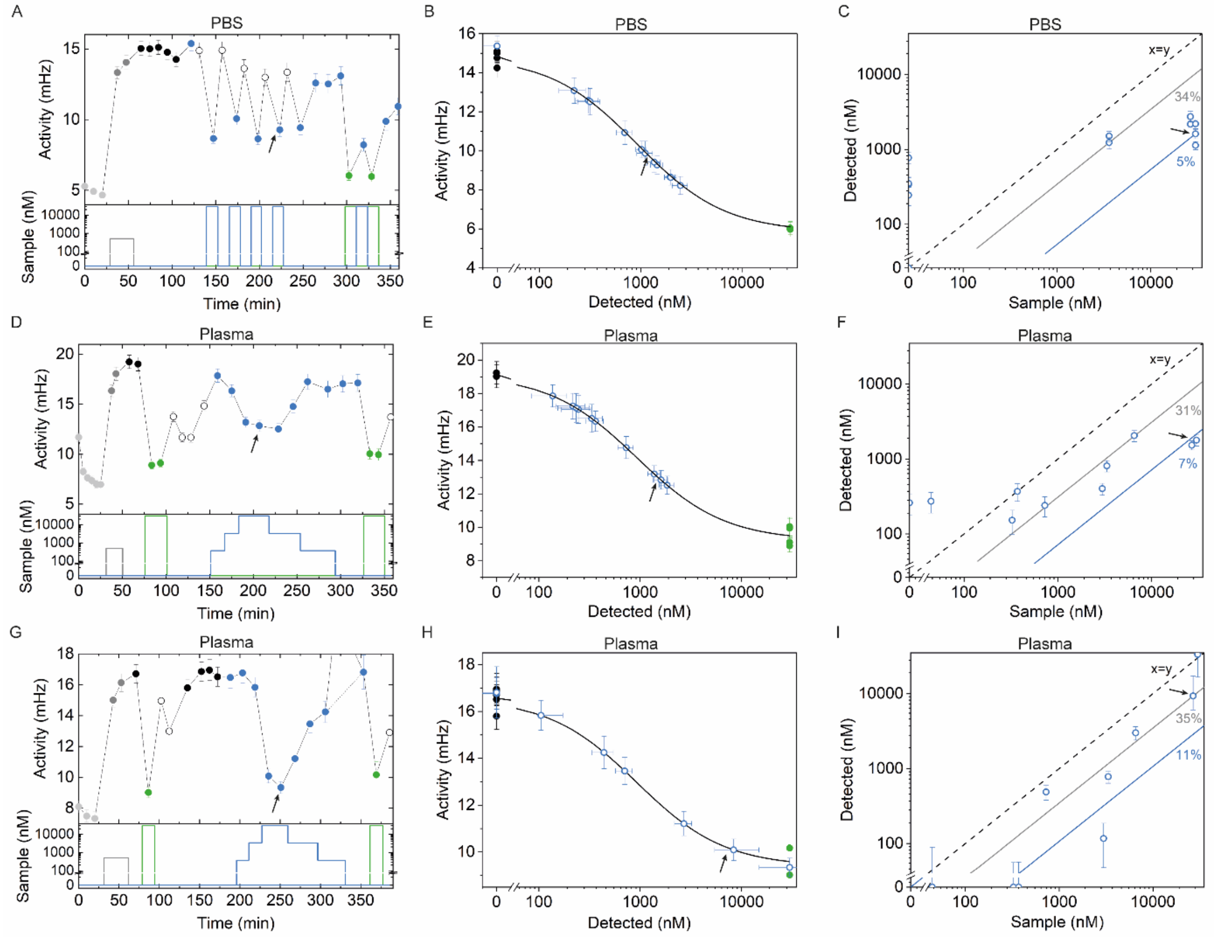

Figure S7: Three microdialysis + BPM experiments to estimate microdialysis recoveries (A-C: PBS, D-I: blood plasma). ADG) Tethered particles (light gray) were activated with 500 pM cortisol analogue (gray), measurements in buffer (black), 30 µM cortisol in buffer (green), blanks (interim points with buffer, open dots) and microdialysis samples (blue). BEH) sigmoidal curves based on calibration data (black and green points) and an EC_{50} of 928 nM. Activity is plotted as a function of calculated detected concentration (blue open dots) with the x-error based on the error in activity and the slope of the sigmoidal curve. CFI) Detected cortisol concentration plotted against the expected cortisol concentration of the sample. Linear fitting through zero is used to determine the recovery, being ~5% (panel C), ~7% (panel F), and ~11% (panel I). Dashed line indicates 100% recovery and gray line 34% (panel C), 31% (panel F) or 35% (panel I) recovery. Arrows indicate the last measured 30 µM cortisol sample before the cortisol concentration was decreased.

The recovery of a microdialysis probe refers to the ratio between analyte concentration in the dialysate and analyte concentration in the original solution. Recovery values are typically well below 100% (Table S1) due to limited analyte diffusion through the membrane[6–10]. The recovery can be estimated by first calibrating the sensor response; thereafter the calibration data can be used to translate the sensor data measured on the dialysate samples into concentration values (Figure S7). The recovery is estimated to be 7.7±3.1%, with some datapoints indicating recoveries of 30-35%.

Table S1: Literature overview of microdialysis applied for cortisol sampling and the specified recoveries.

Probe	Membrane length (mm)	Pore size (kDa)	Recovery	Perfusate	Sampling site	Cortisol detection	Source
MicroEye PME011	15	9	Undefined	0.9% saline & Arixtra	Intravenous	ELISA	Bhake 2020
CMA 66	30	20		Physiological peripheral fluid	Subcutaneous		
CMA/20	10	20	53.5 ± 9.6%	lactated Ringer's solution	In-vitro (37 °C)	HPLC (probe equilibrated for 1 h before sampling)	Schuck 2004
CMA/60	16						
CMA/12	2	Undefined (20 or 100)	15-40%	ACSF	In-vitro (solution bath at 39°C)	HPLC & RIA	Cook 2001
CMA/20	4	20	7.7±3.1% (up to 35%)	PBS	In-vitro (PBS & blood plasma, 37 °C)	Biosensing by Particle Mobility	this work

SUPPORTING METHODS

Particle Preparation for Antibody Screening. Streptavidin-coated magnetic particles (10 mg/mL, Dynabeads MyOne Streptavidin C1, 65001, Thermo Scientific) were incubated for 30 min with an equal volume of 2 nM of 221 bp dsDNA tether (with 5′ Digoxigenin and 5′ Biotin on either end) with a total volume of 4 µL, on a rotating fin. Subsequently, 2.5 volumes of 10 µM capture oligo (Biotin - 5' - GTG CGG CAG GGG TAA GAC CA - 3') were added and incubated for 30 min on a rotating fin, followed by the addition of 50 volumes of 100 µM biotin-PEG (PG1-BN-1k, Nanocs). The particle mixture was washed with 250 volumes of 0.05% Tween20 in PBS, and the particles were reconstituted in 100 volumes of 1% BSA in PBS using magnetic separation and incubated for 30 min. Finally, the particle mixture was sonicated with 10 pulses at 70% with 0.5 duty cycle (Hielscher, Ultrasound Technology).

Substrate Preparation for Antibody Screening. Glass slides (25 x 75 mm, #5, Menzel-Gläser) were cleaned by 10 min of sonication in methanol, after which the glass substrate was dried with a nitrogen stream. A custom-made fluid cell sticker (Grace Biolabs) was attached to the glass slide and the fluid cell was filled with 30 µL PBS. Next, 50 ng/ml anti-digoxigenin antibody (ab76907, Abcam) was aspirated into the system and incubated for 30 min. Then, 25 to 100 µg/ml of antibody F4P1A3 (Cat #MA5-14676, Thermo Scientific), C42 (Cat #MA1-83088, Thermo Scientific), or C53 (Cat #MA1-83090, Thermo Scientific) was added and incubated for 30 min, followed by 30 µL of 1% BSA for 30 min.

Sensor Assembly and Measurements. The fluid cell was filled with 30 µL of particle mixture and incubated for 30 to 45 min to allow particles to sediment to the substrate and attach to the DNA tethers. Thereafter the slide was reversed to allow untethered particles to sediment away from the functionalized surface. Activation of the system was done by adding 30 µL of 100-500 pM cortisol-DNA (analogue) and incubated for 20 min, after which 30 µL of buffer was added. Particle motion was recorded for 5 min.

Danazol. Danazol stock was prepared by dissolving 5 mg/ml in ethanol (technical grade) and diluted further in 0.5 M NaCl/PBS to 30 µM.

REFERENCES

1. Martens, K. J. A., Bader, A. N., Baas, S., Rieger, B. & Hohlbein, J. Phasor based single-molecule localization microscopy in 3D (pSMLM-3D): An algorithm for MHz localization rates using standard CPUs. *J. Chem. Phys.* **148**, (2018).
2. Bergkamp, M. H., Van IJzendoorn, L. J. & Prins, M. W. J. Real-Time detection of state transitions in stochastic signals from biological systems. *ACS Omega* **6**, 17726–17733 (2021).
3. Yan, J., Van Smeden, L., Merkx, M., Zijlstra, P. & Prins, M. W. J. Continuous Small-Molecule Monitoring with a Digital Single-Particle Switch. *ACS Sensors* **5**, 1168–1176 (2020).
4. Lubken, R. M., De Jong, A. M. & Prins, M. W. J. Multiplexed Continuous Biosensing by Single-Molecule Encoded Nanoswitches. *Nano Lett.* **20**, 2296–2302 (2020).
5. Lubken, R. M., de Jong, A. M. & Prins, M. W. J. How Reactivity Variability of Biofunctionalized Particles Is Determined by Superpositional Heterogeneities. *ACS Nano* **15**, 1331–1341 (2021).
6. Plock, N. & Kloft, C. Microdialysis - Theoretical background and recent implementation in applied life-sciences. *Eur. J. Pharm. Sci.* **25**, 1–24 (2005).
7. Shippenberg, T. S. & Thompson, A. C. Overview of Microdialysis. in *Current Protocols in Neuroscience* Chapter 7.1 (2001). doi:10.1002/0471142301.ns0701s00.
8. Bhake, R., Russell, G. M., Kershaw, Y., Stevens, K., Zaccardi, F., Warburton, V. E. C., Linthorst, A. C. E. & Lightman, S. L. Continuous free cortisol profiles in healthy men: Validation of microdialysis method. *J. Clin. Endocrinol. Metab.* **105**, E1749–E1761 (2020).
9. Schuck, V. J. A., Rinas, I. & Derendorf, H. In vitro microdialysis sampling of docetaxel. *J. Pharm. Biomed. Anal.* **36**, 807–813 (2004).
10. Cook, C. J. Measuring of extracellular cortisol and corticotropin-releasing hormone in the amygdala using immunosensor coupled microdialysis. *J. Neurosci. Methods* **110**, 95–101 (2001).
